# Reversible Induction of Phantom Auditory Sensations through Simulated Unilateral Hearing Loss

**DOI:** 10.1371/journal.pone.0035238

**Published:** 2012-06-04

**Authors:** Roland Schaette, Charlotte Turtle, Kevin J. Munro

**Affiliations:** 1 Ear Institute, University College London, London, United Kingdom; 2 School of Psychological Sciences, University of Manchester, Manchester, United Kingdom; University of Sheffield, United Kingdom

## Abstract

Tinnitus, a phantom auditory sensation, is associated with hearing loss in most cases, but it is unclear if hearing loss causes tinnitus. Phantom auditory sensations can be induced in normal hearing listeners when they experience severe auditory deprivation such as confinement in an anechoic chamber, which can be regarded as somewhat analogous to a profound bilateral hearing loss. As this condition is relatively uncommon among tinnitus patients, induction of phantom sounds by a lesser degree of auditory deprivation could advance our understanding of the mechanisms of tinnitus. In this study, we therefore investigated the reporting of phantom sounds after continuous use of an earplug. 18 healthy volunteers with normal hearing wore a silicone earplug continuously in one ear for 7 days. The attenuation provided by the earplugs simulated a mild high-frequency hearing loss, mean attenuation increased from <10 dB at 0.25 kHz to >30 dB at 3 and 4 kHz. 14 out of 18 participants reported phantom sounds during earplug use. 11 participants presented with stable phantom sounds on day 7 and underwent tinnitus spectrum characterization with the earplug still in place. The spectra showed that the phantom sounds were perceived predominantly as high-pitched, corresponding to the frequency range most affected by the earplug. In all cases, the auditory phantom disappeared when the earplug was removed, indicating a causal relation between auditory deprivation and phantom sounds. This relation matches the predictions of our computational model of tinnitus development, which proposes a possible mechanism by which a stabilization of neuronal activity through homeostatic plasticity in the central auditory system could lead to the development of a neuronal correlate of tinnitus when auditory nerve activity is reduced due to the earplug.

## Introduction

Tinnitus is a phantom auditory sensation, the perception of a sound in the absence of a corresponding sound source. In most cases, tinnitus is associated with hearing loss [1], [2], but whether hearing loss actually causes tinnitus has not been clarified yet. Animal models have demonstrated that cochlear damage can lead to behavioural evidence for tinnitus [3], [4] and to the development of increased spontaneous activity of neurons in the central auditory system (see [5] for a review), which has been interpreted as a putative neuronal correlate for the phantom auditory sensation [6]. However, which mechanisms in the brain trigger the development of tinnitus has remained largely unclear.

Computational modelling studies have recently illustrated how cochlear damage might lead to the development of increased spontaneous neuronal activity as observed in animal models of tinnitus [7], [8]. In the models, the development of neural correlates of tinnitus constitutes a side-effect of a stabilization of the mean activity in central auditory neurons through homeostatic plasticity: hearing loss reduces AN activity with a concomitant reduction in excitatory drive to the central auditory system. When homeostatic mechanisms restore average neuronal activity to normal levels by increasing excitation and reducing inhibition, the resulting increase in response gain in the central auditory system causes an amplification of spontaneous neural activity, leading to spontaneous neuronal hyperactivity and the generation of a tinnitus percept. The model’s prediction of tinnitus frequencies closely match subjects’ perceived tinnitus pitch [9]. It follows from the model that reduction of auditory nerve activity, and not cochlear damage as such, might be the determining factor for the development of tinnitus.

It has been long known that complete auditory deprivation can induce tinnitus-like phantom sounds in healthy, normal-hearing volunteers [10], [11]. After several minutes of complete silence in an anechoic chamber or sound-proof booth, participants reported auditory sensations, and the descriptions of the sounds were very similar to those given by tinnitus patients. After leaving the anechoic chamber, the phantom sounds disappeared. This suggests that normal mechanisms of adaptation and plasticity in the brain could also be involved in the development of tinnitus. However, complete auditory deprivation presents a rather drastic scenario, corresponding to profound deafness. It has not been explored yet whether partial deprivation similar to a mild-to-moderate hearing loss typically associated with natural ageing or noise damage, also leads to the perception of phantom sounds. In our previous studies on the effects of unilateral auditory deprivation through an earplug on acoustic reflex thresholds, participants often complained about ringing in their ears after wearing the earplug continuously for several days. We thus studied the occurrence of phantom sounds in 18 healthy volunteers without hearing loss who wore an earplug in one ear for 7 days.

## Methods

This study was approved by the ethics committee of the University of Manchester, and all participants gave written informed consent. The investigation of phantom sounds after earplug usage was embedded in a study on changes in acoustic reflex thresholds and perceived loudness through earplugs (which were assessed on day 1, 7, 8 and 14). 18 volunteers (age range 20–28 years, mean age 23.5±0.44 years; 11 female) were recruited into the study. Participants were required to have normal hearing, i.e. thresholds of <20 dB HL from 0.25 kHz to 8 kHz, and no asymmetry >10 dB between ears at any frequency. A short health questionnaire was used to screen for other conditions, and persons reporting tinnitus were excluded from the study. Pure tone audiometry was performed with an Aurical clinical audiometer and TDH-39 supra-aural headphones. Hearing thresholds were tested at 0.25, 0.5, 1, 2, 4 and 8 kHz. Normal middle ear function was ensured through tympanometry using a GSI TympStar middle ear analyser; participants were required to have middle ear pressure between +50 and −50 daPa and middle ear compliance of 0.3 to 1.6 cm^3^. All equipment was calibrated to accepted standards.

Participants wore a silicone putty ear plug in one ear for 7 days. Each participant was randomly assigned to plug either the left or the right ear, and the participants were instructed to wear the earplug continuously. Sound attenuation of the earplug i.e., the difference in ear canal sound level with and without the earplug in situ, was measured using a clinical probe tube microphone system and a broadband signal of 75 dB SPL. This method was also used to confirm that participants fitted the earplug with a maximum attenuation difference of 3 dB at 1 kHz and 2 kHz when fitting it themselves. At the end of the session, participants were given an “earplug logbook” to record earplug usage (expected to be continuous except for removal for cleaning). They were also told that there might be a possibility of experiencing phantom sounds during earplugs usage, and they were asked to take a note about their occurrence in the logbook. We deliberately did not mention tinnitus in all explanations about phantom sounds to avoid this strongly suggestive term.

All participants who perceived a phantom sound on day 7 underwent a characterisation of the phantom sound using a modified version of the tinnitus spectrum approach [12]. Comparison sounds (pure tones of 0.5, 1, 2, 4, 8, 12, and 16 kHz) were generated using custom-made MATLAB software and were presented to the control ear via Sennheiser HD650 headphones. The comparison tones where first matched to the loudness of the phantom sound using a single-interval adaptive procedure [13]. During loudness matching, sound intensity was limited to levels ≤100 dB SPL. After loudness matching, each sound was presented three times, in random order, and participants were asked to rate the similarity between the pitch of the comparison sound and their phantom sound on a scale of 0–10, with 0 for “completely different” and 10 for “extremely similar”. For comparison tones of 16 kHz, we could not achieve a loudness match for all participants, and thus the results for this frequency were excluded from further analysis.

### Computational Model

To model the development of phantom sounds after earplug-induced auditory deprivation, we employed a computational model of tinnitus development comprising the first stages of auditory processing [7], [8], [9]. The model was organised in frequency channels, with 10 channels per octave. In each channel, we modelled the probability distribution of sound intensities (in units of decibels) by a Gaussian distribution with a mean intensity of 40 dB HL and a standard deviation of 25 dB. The average population response of the auditory nerve fibres in each frequency channel was then modelled by a population rate-intensity function 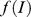 that was assumed to be tuned to the sound intensity distribution (see [7], [8], [9] for more details).

The effects of sound attenuation through an earplug providing 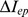 dB attenuation were emulated by shifting the normal AN fibre population response 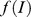 to higher sound intensities:
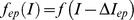
The resulting rate-intensity functions with and without earplugs are shown in Fig. 2b. Using the probability distribution of sound intensities, the effect of sound attenuation on the mean activity of the auditory nerve fibre population was calculated numerically.

The central auditory system stage of the model is based on the circuit that has been proposed for the dorsal cochlear nucleus [14], where projection neurons (PNs) receive inhibition from narrow- (NBI) and wide-band inhibitor (WBI) neurons (Fig. 2a, see [8], [9] for more details). The strengths of these two inhibitory projections, which are controlled by the gain factors 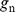 and 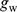, was adjusted such that the projection neurons displayed type III response properties, as we had shown previously that model neurons with these response properties develop tinnitus-related hyperactivity after hearing loss [8]. The mean firing rates of the model neurons in the central auditory system stage of the model, and how they were changed by the earplug, were determined numerically.

Homeostatic plasticity was implemented in the model by simultaneously scaling the strength of the excitatory and inhibitory synapses onto principal neurons in opposite directions [15], [16]. In the model, homeostatic plasticity was assumed to act in PNs in the cochlear nucleus (see [7], [8], [9] for more details on how homeostasis was implemented in the model). Homeostatic plasticity was assumed to stabilize a certain target level of the mean activity, which we defined as the mean activity of a PN receiving input from an unplugged ear and an undamaged cochlea. The change in excitation and inhibition required to restore the mean activity to its target level during earplug-induced auditory deprivation was determined numerically.

Data analysis and model simulations were performed using MATLAB (The MathWorks Inc., Natick, Massachusetts). All error bars are given as ± s.e.m.

## Results

**Figure 1:**
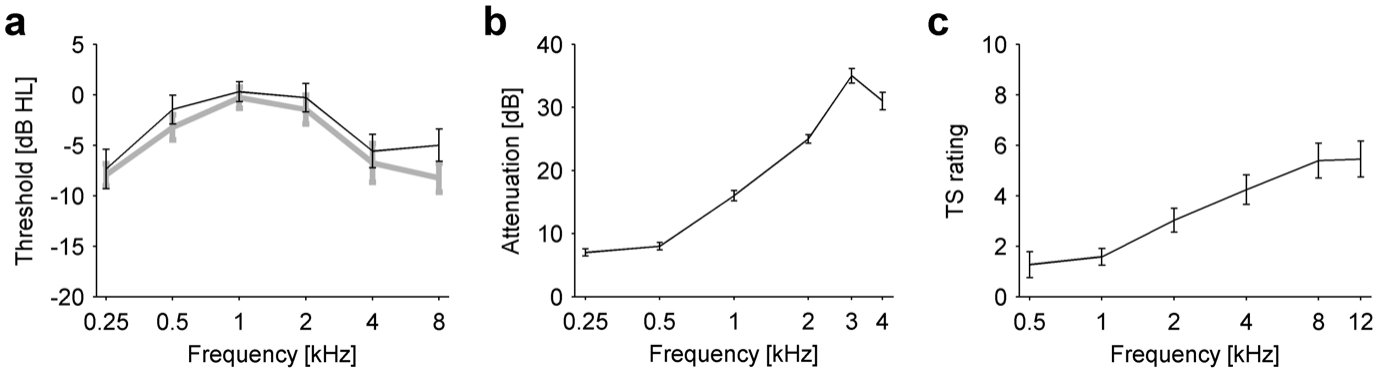
Audiograms, earplug attenuation characteristics, and characterization results for the phantom auditory sensations. **a**) Mean pure tone hearing thresholds (in dB hearing level) for left (grey line) and right ears (black line) of all participants (n = 18). All error bars denote ± s.e.m. **b**) Mean attenuation provided by the earplugs (n = 18). **c**) Mean tinnitus spectrum (TS) rating of the participants who perceived a stable phantom sound on day 7 (n = 11).

18 participants with normal hearing (mean hearing thresholds are shown in Fig. 1a) wore an earplug in one ear for 7 days. The earplugs provided attenuation similar to a mild high-frequency hearing loss, mean attenuation increased from <10 dB at 0.25 kHz to >30 dB at 3 and 4 kHz (Fig. 1b). Fourteen participants reported phantom sounds during the 7 days of earplug usage. The descriptions of the phantom sounds can be found in Table 1. Eleven participants reported phantom sounds while still wearing the earplug on day 7. In these participants, detailed characterisations of the phantom sounds were obtained using the tinnitus spectrum method [12]. The mean tinnitus spectra showed that phantom sounds were generally high-pitched; the highest similarity ratings were obtained for comparison tones at 8 and 12 kHz (Fig. 1c). After the earplug was taken out, the phantom sounds disappeared in all participants. At the end day 7, four participants reported still being aware of a sound in the previously-plugged ear. Only one participant reported hearing phantom sounds on day 8, and no participant reported phantom sounds on day 14.

**Table 1:** Occurrence and description of phantom auditory sensations (PAS).

Subject number	Occurrence of PAS during the week	Description of PAS	PAS present on day 7 for tinnitus spectrum
1	Yes	Fluctuating tone	Yes
2	Yes	Ringing	Yes
3	Yes	Ringing/tones	Yes
4	Yes	Low-pitched buzz	No
5	Yes	Humming & low ringing	Yes
6	Yes	Ringing	Yes
7	Yes	Ringing	Yes
8	Yes	Trains and whistles first, then ringing	No
9	Yes	Humming	No
10	No	/	/
11	No	/	/
12	Yes	Ringing and a high-pitched beep	Yes
13	No	/	/
14	Yes	Humming, cracking, ringing	No
15	No	/	/
16	Yes	High-pitched	Yes
17	Yes	Ringing	Yes
18	Yes	High-pitched buzz	Yes

**Figure 2:**
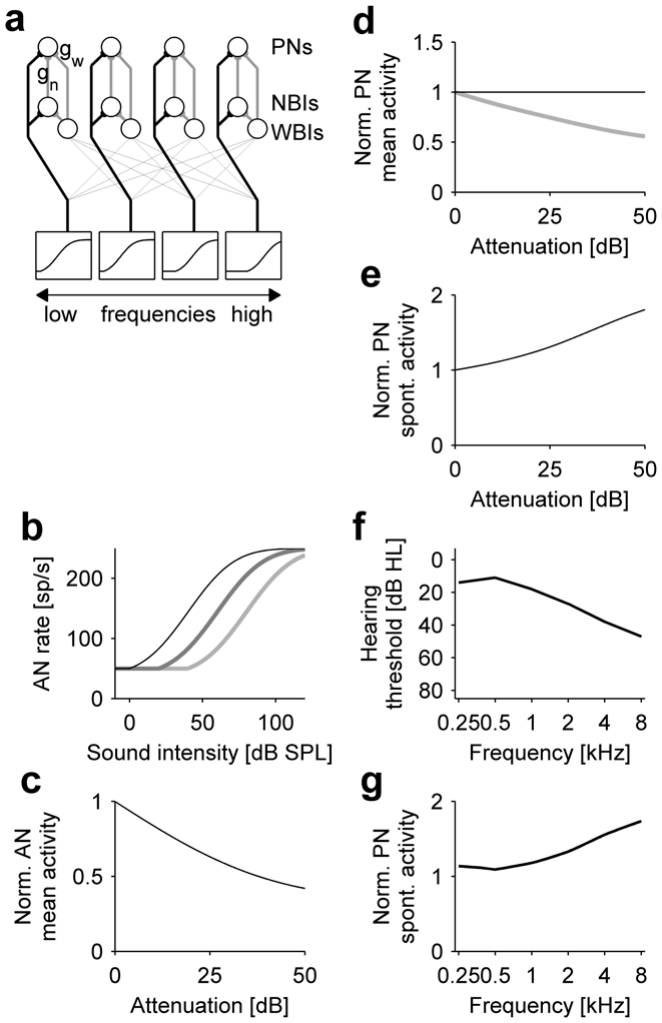
A computational model illustrates how attenuation through an earplug could lead to the development of a neural correlate of phantom sounds. **a**) Architecture of the model covering auditory nerve (bottom) and cochlear nucleus (middle) with projection neurons (PNs), narrow- (NBIs) and wide band inhibitor neurons (WBIs), 4 frequency channels are shown. Circles denote neurons, black lines excitatory and grey lines inhibitory connections. The strength of inhibition from WBIs and NBIs onto the PNs is determined by the gain factors g_w_ and g_n_. **b**) Attenuation through an earplug is modelled by shifting AN rate-vs.-intensity functions to higher intensities, two different degrees of attenuation are shown (black line – normal, dark grey line –20 dB attenuation, light grey line –40 dB attenuation). **c**) The mean AN activity is reduced in proportion to the degree of attenuation. **d**) Attenuation reduces the mean activity of the principal neurons in the CN stage of the model (grey line). By increasing excitation and decreasing inhibition, homeostatic plasticity is able to restore the mean activity to its healthy target level (black line). **e**) As a side-effect of activity stabilization through homeostatic plasticity, spontaneous firing rates in the model PNs are increased in dependence upon the degree of attenuation. **f**) Average hearing thresholds of our participants with the earplug in place. **g**) After homeostatic plasticity has compensated for the earplug-induced decrease in mean activity, the PNs in the CN stage of the model display a pattern of increased spontaneous activity in the high frequency range.

In order to understand how attenuation through an ear-plug could lead to the occurrence of phantom sounds, we employed a computational model of the first stages of auditory processing (Fig. 2a) that we had originally developed to model tinnitus-related changes in the auditory brainstem after cochlear damage [7], [8]. Here, only one additional parameter was introduced to the model in order to capture the effects of sound attenuation through the earplug by shifting the auditory nerve rate-vs.-intensity functions to higher sound intensities (Fig. 2b, see also Methods). All parameters of the model relating to the central auditory pathways remained unchanged to previous versions of the model [7], [8]. In a first run of simulations, we only considered “model earplugs” with a flat attenuation profile, i.e. the degree of attenuation was identical for all frequency channels, varying attenuation from 0 to 50 dB. We found that attenuation reduced the mean activity in both the AN (Fig. 2c) and the central auditory system stage of the model (Fig. 2d, grey line), and the degree of activity reduction was proportional to the attenuation provided by the earplug.

In the model, we assumed that the mean activity levels of principal neurons (PNs) in the central auditory system were stabilized by homeostatic plasticity. After the model’s ear had been plugged, homeostatic plasticity thus tried to compensate for the decrease in PN activity by increasing excitation and decreasing inhibition. When homeostasis restored the mean activity of the PNs in the model cochlear nucleus to the healthy target level (Fig. 2d, black line), the resulting increase in neuronal response gain also caused an over-amplification of spontaneous input from the auditory nerve, leading to increased spontaneous firing rates in the cochlear nucleus PNs (Fig. 2e). Such hyperactivity could be a neural substrate for the perception of phantom sounds. The degree of elevation of the spontaneous activity was proportional to the amount of attenuation provided by the earplug (Fig. 2e). To directly relate the model to our experimental results, we evaluated its prediction for the mean hearing threshold curve of our participants with the ear plug in place (Fig. 2f). This curve was estimated by adding the mean attenuation values of the earplugs (Fig. 1b) to the mean hearing threshold curve (Fig. 1a). As the attenuation value for 8 kHz could not be measured due to technical limitations, we estimated it based on data from a previous study [17]. For auditory deprivation as induced by the earplugs, the model predicted a hyperactivity profile that peaked at high frequencies (Fig. 2g), thus showing a pattern similar to the ‘tinnitus spectra’ obtained for the phantom sounds experienced by our participants (Fig. 1c). Finally, also in the model the development of phantom sounds was fully reversible, spontaneous firing rates decreased back to the healthy normal level when the “model earplug” was taken out.

## Discussion

Our results demonstrate that unilateral auditory deprivation similar to a mild high-frequency hearing loss is sufficient to induce phantom auditory sensations in healthy normal hearing subjects without tinnitus. This suggests that auditory deprivation (not necessarily involving cochlear damage), and the concomitant reduction of auditory nerve activity, could be the dominant factor for the development of phantom sounds. The characteristics of the phantom auditory sensations perceived by our participants were very similar to tinnitus: Firstly, the descriptions of the phantom sounds resembled those of tinnitus sounds given by tinnitus patients [2]. Secondly, the mean tinnitus spectrum reached its highest values at 8 and 12 kHz, which is similar to tinnitus spectra of tinnitus patients with mild hearing loss [18]. Using a computational model, we have illustrated how plasticity mechanisms that stabilize neuronal activity levels in the central auditory system could lead to the development of a neural correlate of tinnitus during auditory deprivation.

The participants in our study wore earplugs in one ear only. Bilateral earplugs were not tested due to ethical considerations, as plugging both ears could increase the probability of dangerous situations, e.g. in traffic. Thus, the phantom auditory sensations perceived by our participant could have developed in reaction to decreased input from the ipsilateral ear, the imbalance between the ears, or both. Given that binaural deprivation in a sound-proof booth also gives rise to phantom sounds [10], [11], we would expect to obtain similar results when both ears are plugged.

In the recruitment information sheet for the study, our participants were informed that they might experience phantom sounds while their ear was plugged, and this suggestion itself might have increased the incidence of phantom sounds, e.g. by focussing attention. One way of addressing this problem would be to use a control group wearing non-attenuating earplugs. However, a non-attenuating earplug can be easily recognized by the wearer. Alternatively, a future study could employ earplugs with different degrees of attenuation to study whether the occurrence of phantom sounds depends on the degree of attenuation, which was beyond the means of our current study. Moreover, it also remains to be shown if a similar relation between phantom sound characteristics and attenuation profile can also be obtained when only low frequencies are attenuated, which would support a true frequency-specific adaptation effect. However, we believe that the fact that the phantom sounds were stable enough to run a full tinnitus spectrum characterisation in 11/18 participants, that their pitch generally matched the attenuation characteristics of the earplugs, and that the phantom sounds disappeared in all participants after removing the earplug, already support a causal relation between plugging the ear and the occurrence of phantom sounds.

Several mechanisms might contribute to the occurrence of phantom sounds while the ear is plugged: Firstly, an intrinsic tinnitus could have been unmasked through the reduction of AN input caused by the earplug. However, as the earplug does not change the spontaneous activity of the auditory nerve, and thus not the neural activity coding for silence, changes in the central auditory system would still be required to give rise to a conscious tinnitus percept. Secondly, the neuronal response gain in the central auditory system could have been increased in response to the auditory deprivation. The resulting over-amplification of spontaneous activity in this scenario could account for the occurrence of phantom sounds. It has been shown recently that earplug-induced unilateral auditory deprivation lowers the acoustic reflex threshold in the ipsilateral ear, a physiological change indicative of increased gain in the auditory brainstem [17]. A putative mechanism for increased gain has been demonstrated in animal experiments: plugging one ear for 24 hours lead to an increase in excitation and a decrease in inhibition in the cochlear nucleus, both of which were completely reversible [19]. These plastic changes would be consistent with the activation of homeostatic plasticity, a mechanism that is believed to stabilize the mean neuronal activity on time scales of hours to days by scaling the strength of excitatory and inhibitory synapses and regulating intrinsic neuronal excitability [16], [20], [21].

Homeostatic plasticity is also a key factor in our computational model for the development of tinnitus [7], [9]. Homeostatic plasticity and similar gain adaptation mechanisms have also been explored in other models of tinnitus generation [22], [23], which could therefore be expected to give similar predictions for the effects of earplugs. It follows from our model that earplugs could also induce phantom sounds (Fig. 2), as verified experimentally in this study. However, not all participants perceived phantom sounds while their ears were plugged, even though our model would always predict an increase in spontaneous activity in the auditory brainstem. This discrepancy can be understood in the light of a recent theory of tinnitus, which postulates that pathological changes in spontaneous neuronal activity in the auditory brainstem are only a pre-requisite for tinnitus, whereas conscious perception of tinnitus requires additional changes at the cortical level and a failure of thalamic gating mechanisms [24]. As not all of our participants perceived ‘tinnitus’ during earplug usage, the putative mechanisms involved in the gating of a ‘brainstem tinnitus’ could be studied using the earplug paradigm.

When auditory nerve activity was ‘renormalized’ by removing the earplug, the phantom auditory sensations disappeared in all participants. This is consistent with the mechanism of activity stabilization in our model. Thus, one might conclude that tinnitus (occurring in association with hearing loss) could be decreased by renormalizing or simply increasing AN activity through increased acoustic stimulation, delivered for example through a hearing aid. However, there are technical and biological limitations for the renormalization of auditory nerve activity after cochlear damage. Firstly, the limited frequency range of current ear-level devices restricts the frequency range where AN activity can be increased through amplification, and we have shown recently that this factor influences the effectiveness of tinnitus management through hearing aids and noise generators [25]. Secondly, severe damage to the cochlear transducing cells or/and associated neurones, i.e. cochlear dead regions [26], will often preclude a complete renormalization of auditory nerve activity by means of acoustic stimulation. In these cases, electrical stimulation of the remaining auditory nerve fibres might be a more promising option. We hope that our demonstration that the perception of phantom sounds can be triggered by a reduction of auditory nerve activity independent of cochlear damage will open up new perspectives for the understanding of phantom auditory sensations and inspire new approaches for the treatment of tinnitus.
